# Population-level benefits of increasing influenza vaccination uptake among Italian older adults: results from a granular panel model

**DOI:** 10.3389/fpubh.2023.1224175

**Published:** 2023-08-03

**Authors:** Alexander Domnich, Andrea Orsi, Donatella Panatto, Matilde Ogliastro, Alessandra Barca, Fabrizio Bert, Danilo Cereda, Maria Chironna, Claudio Costantino, Daniel Fiacchini, Elena Pariani, Caterina Rizzo, Enrico Volpe, Giancarlo Icardi, The FluCoV Study Group, Daniela Amicizia

**Affiliations:** ^1^Hygiene Unit, San Martino Policlinico Hospital-IRCCS for Oncology and Neurosciences, Genoa, Italy; ^2^Department of Health Sciences (DISSAL), University of Genoa, Genoa, Italy; ^3^Interuniversity Research Center on Influenza and Other Transmissible Infections (CIRI-IT), Genoa, Italy; ^4^Directorate for Health and Social Policy, Lazio Region, Rome, Italy; ^5^Department of Public Health and Pediatrics, University of Turin, Turin, Italy; ^6^Hygiene and Infection Control Unit, ASL TO3, Turin, Italy; ^7^Directorate General for Health, Lombardy Region, Milan, Italy; ^8^Department of Interdisciplinary Medicine, University of Bari, Aldo Moro Policlinico, Bari, Italy; ^9^Department of Health Promotion Sciences, Maternal and Infant Care, Internal Medicine and Medical Specialties (PROMISE) “G. D'Alessandro, ” University of Palermo, Palermo, Italy; ^10^Public Health Department, AST Ancona, Ancona, Italy; ^11^Department of Biomedical Sciences for Health, University of Milan, Milan, Italy; ^12^Department of Translational Research on New Technologies in Medicine and Surgery, University of Pisa, Pisa, Italy

**Keywords:** influenza, vaccination, vaccine impact, older adults, modeling, Italy

## Abstract

**Background:**

The impact of seasonal influenza vaccination (SIV) on mortality is still controversial; some studies have claimed that increasing vaccination coverage rates is beneficial, while others have found no significant association. This study aimed to construct a granular longitudinal dataset of local VCRs and assess their effect on pneumonia- and influenza-related (P&I) mortality among Italian adults aged ≥ 65 years.

**Methods:**

NUTS-3 (nomenclature of territorial units for statistics) level data on SIV coverage were collected via a survey of local data holders. Fixed- and random-effects panel regression modeling, when adjusted for potential confounders, was performed to assess the association between local SIV coverage rates and P&I mortality in older adults.

**Results:**

A total of 1,144 local VCRs from 2003 to 2019 were ascertained. In the fully adjusted fixed-effects model, each 1% increase in vaccination coverage was associated (*P* < 0.001) with a 0.6% (95% CI: 0.3–0.9%) average over-time decrease in P&I mortality. With an annual average of 9,293 P&I deaths in Italy, this model suggested that 56 deaths could have been avoided each year by increasing SIV coverage by 1%. The random-effects model produced similar results. The base-case results were robust in a sensitivity analysis.

**Conclusion:**

Over the last two decades, Italian jurisdictions with higher SIV uptake had, on average, fewer P&I deaths among older adults. Local policy-makers should implement effective strategies to increase SIV coverage in the Italian senior population.

## 1. Introduction

Compared with other age-classes, older adults display a disproportionately high risk of severe influenza outcomes (1). Annual vaccination is the best way to prevent seasonal influenza, and older adults are the primary target group for seasonal influenza vaccination (SIV) (2, 3).

The effects of SIV on influenza-related endpoints can be established by evaluating: (i) vaccine efficacy (risk reduction resulting from vaccination, as measured in randomized controlled trials); (ii) vaccine effectiveness (risk reduction resulting from vaccination, as measured in observational studies) and (iii) vaccine impact (reduction in the incidence of influenza-related outcomes in a population in which some subjects are vaccinated, as measured through modeling) (4). Although randomized studies are the gold standard technique for evaluating vaccine-induced protection, their external validity may be poor, since some population strata (e.g., the oldest old, subjects with multimorbidity or polypharmacy) are usually systematically excluded (5, 6). In this regard, well-designed observational studies can address the issue of inclusiveness, thereby complementing experimental evidence (5). On the other hand, both experimental and observational studies are usually conducted within a limited time-span (typically 1–3 seasons) that is characterized by specific patterns of viral circulation and degrees of vaccine match. For instance, across the last 18 seasons, overall SIV effectiveness in the United States (US) ranged from 10% (7) to 60% (8). From the policy-makers' perspective, it should be stressed that VE estimates alone are not a measure of the full population benefit of SIV and should be interpreted contextually (9). In this regard, modeling studies on vaccine impact may further support policy decisions.

The benefits of SIV on mortality in older adults is an ongoing controversy (10). While several time-series analyses, which attributed ~5% of all winter deaths to influenza, failed to demonstrate a decline in influenza-related mortality on increasing the vaccination coverage rate (VCR), some cohort studies have reported a paradoxically high (~50%) relative reduction in the total risk of winter mortality. Overestimation of the effect of SIV may be driven by both the frailty selection bias and the use of non-specific outcomes, such as all-cause (AC) mortality (10). Similarly, previous Italian modeling studies (11, 12) have reached different conclusions. Rizzo et al. (11) did not find a significant decrease in pneumonia- and influenza-related (P&I) and AC excess mortality following an increase in SIV coverage rates over 31 influenza seasons. Despite its long period of observation, that study (11) did not consider large between-region differences in both socio-structural dynamics and SIV policies. A later study by Fallani et al. (12) attempted to overcome the above-mentioned shortcomings by analyzing a panel dataset in which 21 Italian regions were monitored over 7 seasons. The authors concluded that each 1% increase in SIV uptake by older adults was associated (*P* < 0.001) with a 1.6–1.9% decrease in P&I mortality. However, while panel data are better able to capture unobserved effects than the analysis of historical trends (13), the study by Fallani et al. (12) was limited by its low number of observations in both time-series and cross-sectional components of the panel.

The ecological and evolutionary dynamics of infections like influenza play out over a broad range of interconnected temporal, spatial and organizational dimensions (14). Influenza modeling and trend assessment can further integrate the individual-level experimental and observational evidence by assessing the average impact of SIV campaigns on the incidence of influenza-related outcomes when complex spatio-temporal, socio-economic and policy-related factors are taken into account (14, 15). By establishing a granular space-time dataset, the present study aimed to assess the locally varying dynamic association between SIV uptake and influenza-related mortality in the Italian older population.

## 2. Methods

### 2.1. Overall study design

In this ecological study, we adopted an econometric panel data approach, in which each cross-sectional spatial unit was tracked over time. The study period was determined by the availability of outcome data, and covered 17 consecutive years (from 2003 to 2019). Years from 2020 onwards were not considered, owing to the impact of COVID-19 on both P&I mortality and influenza virus circulation. The spatial unit considered in the study was a single Province/Metropolitan City (*N* = 103/110), which is equivalent to the lowest Eurostat NUTS-3 (nomenclature of territorial units for statistics) level (16). Indeed, modeling at a higher level of spatial granularity allows to capture previously unseen associations or to uncover finer details of the known relationships. By incorporating time trend and sufficiently granular spatial components, panel datasets are able to dramatically increase the sample size and to identify and measure effects that are not detectable in pure time-series or cross-sectional data (13).

Data on P&I mortality and potential confounders were taken from publicly available sources (17–20), while NUTS-3 level VCRs among individuals aged ≥ 65 years (VCR_65+_) were established *ad hoc* (see below). NUTS-3 data for both outcomes and confounders were available for all study years. If NUTS-3 VCR_65+_ data were not available in a region or not routinely collected, the previous NUTS-2 level data on VCR_65+_ (21), outcomes and confounders were used. Indeed, imputing missing NUTS-3 VCR_65+_ data (both missing years and missing provinces) was judged unfeasible.

### 2.2. Study population

The study population was composed of Italian adults aged ≥ 65 years. During the whole study period, SIV was recommended and fully reimbursed for all subjects aged ≥ 65 years (22).

### 2.3. Study outcome

The study endpoint was the P&I mortality rate (per 10,000) among adults aged ≥ 65 years (P&I_65+_) observed in a NUTS-3 location *i* during the year *t* and derived from death certificates codified according to the international classification of diseases (ICD) (ICD-8: 470–474 and 480–486; ICD-9: 487 and 480–486; ICD-10: J09–J11 and J12–J18 for influenza and pneumonia, respectively) (17).

### 2.4. Study variables and potential confounders

The Italian Ministry of Health routinely collects and reports (21) VCR_65+_ on the NUTS-2 level in 19 Regions and two Autonomous Provinces of Trento and Bolzano only. To obtain the more granular NUTS-3 VCR_65+_ (% of older adults vaccinated), a search of local datasets, statistical compendiums and other relevant documents was first conducted. Potential local data holders were then contacted and asked to provide these statistics for the longest possible time-span.

The list of potential confounders and effect modifiers was determined through an internal discussion and previous research (11, 12). These included independent variables related to socio-economic, environmental, healthcare and virological domains (Table 1) (17–20).

**Table 1:** Potential confounders and their definitions.

**Group**	**Variable**	**Definition**	**Ref**
Socio-economic	P_75+_	Adults aged ≥ 75 years as a proportion (%) of those aged ≥ 65 years	17
	P_≤ 14_	Children aged ≤ 14 years as a proportion (%) of the total population	17
	GDP	Gross domestic product (1,000 €) per capita based on purchasing power parity	18
	Mort	Baseline mortality rate per 10,000 inhabitants	17
Environmental	Temp	Monthly average temperature (°C) in the month of January (typically the coldest month) registered in the chief town of a given location	19
	Dens	Population density, inhabitants per km^2^	17
Healthcare	Bed	Ordinary hospital beds per 10,000 inhabitants	17
	HCP	Healthcare professionals operating in public and accredited private health facilities per 10,000 inhabitants	17
Virological	H1N1s	Dummy variable for seasons, in which seasonal pre-2009 A/H1N1s subtype was predominant	20
	H1N1pdm09	Dummy variable for seasons, in which A(H1N1)pdm09 subtype was predominant	20
	H3N2	Dummy variable for seasons, in which A(H3N2) subtype was predominant	20
	B	Dummy variable for seasons, in which type B was predominant	20
	H1N1pdm09/H3N2	Dummy variable for seasons, in which A(H1N1)pdm09 and A(H3N2) subtypes were co-dominant	20
	H1N1s/B	Dummy variable for seasons, in which A(H1N1)s and B (sub)types were co-dominant	20

### 2.5. Statistical analysis

The association between P&I_65+_ and VCR_65+_ was examined by applying both fixed-effects (FE) and Swamy-Arora random-effects (RE) models for unbalanced panels implemented in the plm R package (23). The model structure may be presented as follows:


$$\begin{array}{l} \log_{e}{\left(P\&I_{65\text{+}}\right)}_{\text{i},\text{t}}=\beta_{1}\times {\left(VCR_{65\text{+}}\right)}_{\text{i},\text{t}}+\Sigma \left[\beta_{x}\times {\left(Confounders\right)}_{\text{i},\text{t}}\right]\\ \;+\alpha \left(i\right)+\;{\varepsilon_{i}}_{,\text{t}}, \end{array}$$

for *i* = 1…110 and *t* = 2003…2019, where β_1_ and β_*x*_ are regression coefficients; “Confounders” is a set of potential confounders presented in Table 1; α is the unobserved time-invariant local effect (in the FE model) or constant intercept (in the RE model); *i* is a location; *t* is a year; ε is the error term.

The degree of imbalance, which was driven by some missing VCR_65+_, in the base-case multivariable model was low (γ = 0.92; ν = 0.93). The main advantage of the FE models is that they take into account potential bias from omitted time-invariant variables by eliminating between variation from the estimation. On the other hand, RE models may be more efficient; however, if individual effects are correlated with regressors, the RE estimation would be inconsistent (13, 24, 25). Hausman's specification test was used to select between the models (23). For all models, rates that were not percentages were loge-transformed and interpreted as elasticities (e.g., the regression coefficient for VCR_65+_ is a percent change in P&I_65+_ associated with a 1% change in VCR_65+_) (26). Considering the presence of cross-sectional dependence (Pesaran's test: *P* < 0.001) and serial correlation (Breusch-Godfrey/Wooldridge's test: *P* < 0.001), the Driscoll-Kraay robust covariance matrix estimator was used to establish heteroskedasticity and autocorrelation consistent (HAC) standard errors (SEs) and associated *p*-values. Finally, a sensitivity analysis was conducted in which all NUTS-2 units (*N* = 379) were excluded.

All analyses were performed in R stat packages v.4.2.2 (R Foundation for Statistical Computing; Vienna, Austria).

## 3. Results

NUTS-3 VCR_65+_ data were obtained for 14 out of 20 Italian regions, and were available for 7–17 seasons. In summary, a total of 1,144 local VCR_65+_ were included in the analysis (Supplementary Table 1).

Table 2 summarizes the distribution patterns of both the study outcome and independent variables. On univariate FE analysis, each 1% increase in VCR_65+_ was associated with a 1.7% (95% CI: 1.5–2.0%) average over-time decrease in P&I_65+_. The unadjusted RE coefficient was the same. Other statistically significant crude associations included proportions of ≥ 75-year-olds and children, baseline mortality, gross domestic product (GDP), average January temperature, and patterns of circulating viruses (Table 2).

**Table 2:** Summary statistics of the study variables and unadjusted fixed- and random-effects models on the association between pneumonia- and influenza-related mortality among Italian older adults and potential predictors.

**Variable**	***n***	**Mean**	**SD**	**Min**	**Max**	**Estimate (HAC SE) FE model**	**Estimate (HAC SE) RE model**
P&I${}_{65+}^{\text{a}}$	1,523	7.7	3.5	1.3	23.1	–	–
VCR_65+_	1,144	59.3	10.0	29.1	84.7	−0.017 (0.001)^***^	−0.017 (0.001)^***^
P_75+_	1,523	49.9	2.8	41.9	56.1	0.058 (0.001)^***^	0.058 (0.001)^***^
P_≤ 14_	1,523	13.3	1.4	9.6	18.2	−0.110 (0.018)^***^	−0.111 (0.017)^***^
GDP^a^	1,523	25.6	6.3	13.1	50.7	1.676 (0.139)^***^	1.477 (0.091)^***^
Mort^a^	1,523	106.8	15.6	74.5	153.8	3.339 (0.215)^***^	3.025 (0.180)^***^
Temp	1,523	5.6	3.4	−7.7	14.3	0.004 (0.004)	−0.012 (0.005)^*^
Dens^a^	1,523	243.4	250.6	37.1	2083.0	−0.090 (0.476)	0.024 (0.076)
Bed^a^	1,523	34.0	8.5	15.7	73.2	−0.755 (0.101)^***^	−0.574 (0.089)^***^
HCP^a^	1,523	102.3	28.2	21.7	177.9	−0.103 (0.148)	0.127 (0.095)
H1N1s dummy	1,523	0.1	0.2	0	1	Ref	Ref
H1N1pdm09 dummy	1,523	0.1	0.3	0	1	0.080 (0.023)^***^	0.080 (0.023)^***^
H3N2 dummy	1,523	0.5	0.5	0	1	0.199 (0.021)^***^	0.199 (0.021)^***^
B dummy	1,523	0.2	0.4	0	1	0.367 (0.028)^***^	0.367 (0.028)^***^
H1N1s/B dummy	1,523	0.1	0.3	0	1	0.010 (0.030)	0.010 (0.030)
H1N1pdm09/H3N2 dummy	1,523	0.1	0.2	0	1	0.517 (0.027)^***^	0.517 (0.027)^***^

Bed, ordinary hospital beds per 10,000 inhabitants; Dens, population density, inhabitants per km^2^; FE, fixed-effects model; GDP, gross domestic product (1,000 €) per capita based on purchasing power parity; HAC SE, heteroskedasticity and autocorrelation consistent standard errors; HCP, healthcare professionals operating in public and accredited private health facilities per 10,000 inhabitants; Mort, baseline mortality rate per 10,000 inhabitants; P_≤ 14_, proportion (%) of children aged ≤ 14 years in the total population; P_75+_, proportion (%) of older adults aged ≥ 75 years in the population aged ≥ 65 years; P&I_65+_, mortality due to pneumonia and influenza in subjects aged ≥ 65 years; RE, random-effects model; SD, standard deviation; VCR_65+_, influenza vaccination coverage rate in subjects aged ≥ 65 years.

a Regression coefficients are based on log_*e*_-transformed variables.

*** *P* < 0.001.

* *P* < 0.05.

In the fully adjusted analysis (Table 3), the parameter estimates of both the FE and RE models were similar, although Hausman's test suggested (*P* = 0.001) that the RE model was inconsistent. The FE model showed that each 1% increase in VCR_65+_ was associated with a 0.6% (95% CI: 0.3–0.9%) average over-time decrease in P&I_65+_. GDP, baseline mortality and proportion of children were positive predictors of P&I_65+_. Analogously, as compared with seasonal pre-2009 H1N1s subtype, seasons (co)dominated by A(H3N2) and B strains were associated with higher P&I_65+_. By contrast, each 1°C increase in the average January temperature was associated (*P* = 0.001) with a 1.3% decrease in P&I_65+_. The FE and RE models explained 48.2 and 53.4% of variance, respectively (Table 3).

**Table 3:** Multivariable fixed- and random-effects models on the association between pneumonia- and influenza-related mortality and Influenza vaccination coverage rates among Italian older adults.

**Variable**	**FE model**		**RE model**	
	**Estimate (HAC SE)**	***P*** **-value**	**Estimate (HAC SE)**	***P*** **-value**
VCR_65+_	−0.006 (0.001)	< 0.001^***^	−0.007 (0.001)	< 0.001^***^
P_75+_	−0.004 (0.009)	0.66	−0.007 (0.009)	0.45
P_≤ 14_	0.091 (0.025)	< 0.001^***^	0.088 (0.020)	< 0.001^***^
GDP^a^	1.043 (0.191)	< 0.001^***^	1.110 (0.113)	< 0.001^***^
Mort^a^	2.474 (0.285)	< 0.001^***^	2.060 (0.230)	< 0.001^***^
Temp	−0.013 (0.004)	0.001^**^	−0.018 (0.003)	< 0.001^***^
Dens^a^	0.045 (0.409)	0.91	0.030 (0.038)	0.42
Bed^a^	0.021 (0.124)	0.87	0.015 (0.105)	0.88
HCP^a^	−0.127 (0.174)	0.46	−0.054 (0.116)	0.64
H1N1s dummy	Ref	Ref	Ref	Ref
H1N1pdm09 dummy	0.002 (0.036)	0.96	0.013 (0.037)	0.72
H3N2 dummy	0.106 (0.033)	0.002^**^	0.128 (0.031)	< 0.001^***^
B dummy	0.129 (0.040)	0.001^**^	0.156 (0.038)	< 0.001^***^
H1N1s/B dummy	−0.037 (0.044)	0.41	−0.016 (0.042)	0.70
H1N1pdm09/H3N2 dummy	0.142 (0.041)	< 0.001^***^	0.182 (0.038)	< 0.001^***^
*R* ^2^	0.482	< 0.001^***^	0.534	< 0.001^***^

Bed, ordinary hospital beds per 10,000 inhabitants; Dens, population density, inhabitants per km^2^; FE, fixed-effects model; GDP, gross domestic product (1,000 €) per capita based on purchasing power parity; HAC SE, heteroskedasticity and autocorrelation consistent standard errors; HCP, healthcare professionals operating in public and accredited private health facilities per 10,000 inhabitants; Mort, baseline mortality rate per 10,000 inhabitants; P_≤ 14_, proportion (%) of children aged ≤ 14 years in the total population; P_75+_, proportion (%) of older adults aged ≥ 75 years in the population aged ≥ 65 years; RE, random-effects model; SD, standard deviation; VCR_65+_, influenza vaccination coverage rate in subjects aged ≥ 65 years.

a Regression coefficients are based on log_*e*_-transformed variables.

*** *P* < 0.001.

** *P* < 0.01.

In the sensitivity analysis (Supplementary Table 2), the regression output was similar, suggesting the robustness of the base-case model.

## 4. Discussion

Models are undoubtedly useful tools for structured decision-making and enable the principal stakeholders to consider the potential impact of VCRs on pre-defined population health outcomes. Importantly, models are more useful as tools for forecasting the likely pattern of disease-specific endpoints that might emerge under various circumstances over time (27). In this study, we collected a large amount of local SIV VCR_65+_ and used these to populate an econometric model of the impact of SIV over 17 years. Our model showed that each 1% increase in VCR_65+_ was associated with a 0.6% (95% CI: 0.3–0.9%) average over-time decrease in P&I_65+_. During the study period, an annual average of 9,293 P&I deaths in older adults were reported in Italy (17). Translated into absolute numbers, our model suggests that an average of 56 P&I deaths could have been avoided each year by increasing VCR by only 1%.

As mentioned earlier, previous Italian modeling studies (11, 12, 28) on the population-level benefits of increasing SIV VCRs have reached somewhat contrasting conclusions. A cyclic Serfling-type model on 31-year monthly data (1970–2001) implemented by Rizzo et al. (11) failed to demonstrate any marked association between VCRs and P&I/AC excess mortality in adults aged 65–84 years. Specifically, in the period (1970–1986) of low SIV VCR (< 8%) a significant (*P* ≤ 0.002) declining trend in both P&I and AC excess mortality rates over time was discerned. By contrast, in the period (1987–2001) of rapidly increasing SIV uptake, the authors found no evidence of any trend in either P&I (*P* = 0.36) or AC (*P* = 0.75) excess mortality (11). On the other hand, a correlational 12-season (from 2005/06 to 2016/17) study by Manzoli et al. (28) reported a significant (*P* = 0.036) linear increase in the annual incidence of influenza-like illness (ILI) following the registered decline in VCR_65+_. Each one-point increase in VCR_65+_ was associated with an 8.6% (95%: 1.0–16.7%) reduction in the ILI attack rate. In other words, at least 2,690 ILI cases in the Italian senior population could have been prevented by increasing VCR_65+_ by 1% (28). Owing to some methodological similarities, our results are more similar to the longitudinal FE model by Fallani et al. (12), who reported a significant inverse relationship between P&I_65+_ and VCR_65+_. However, the adjusted regression coefficient for VCR_65+_ in our study was about three times lower (−0.006 vs. −0.019), while the unadjusted (−0.017) regression coefficient approached that of Fallani's study (12). This difference is probably ascribable to the fact that the previous model was not corrected for the baseline mortality rate, which alone explained 37.1% of variance in our study. Contrary to our approach, which was based on 1,144 space-time observations, the study by Fallani et al. (12) included only 147 regression points and was limited to the post-2009 pandemic period. In sum, the inconsistencies in both the present and past research (11, 12, 28) are apparently driven by the modeling approach, time period and influenza-related proxy measures used. First, by incorporating intra-country dynamics, panel models have advantages over time-series, as they allow for heterogeneity across spatial units, thereby increasing the reliability of results, may be more efficient, and suffer less from deficient statistical power (13). Second, the relative frequency of influenza-related population-wide indicators may have changed over recent decades. Indeed, evidence from the US (29) has highlighted that, unlike pneumonia caused by *Streptococcus pneumoniae* and *Haemophilus influenzae*, the rates of both hospitalization and case-fatality due to pneumonia caused by influenza virus increased from 2002 to 2011 by 132% (*P* < 0.001) and 67% (*P* < 0.001), respectively. Similarly, during the current study period of 2003–2019, an increasing trend (Mann-Kendall trend test: *P* < 0.001; results not shown) in P&I_65+_ was reported in Italy (17). These observations contrast with those from the earlier time period (1970–2001) investigated in the study by Rizzo et al. (11). Finally, the sensitivity and specificity of influenza-related proxies may have a dramatic impact on SIV effectiveness (4). Indeed, it has been noted (30) that evaluating the benefits of SIV at the population level is challenging, since some disease proxies may lack statistically significant findings.

The main limitation of the present study is that several NUTS-3 observations were missing, especially in southern regions. Ideally, the 17-year dataset of 103/110 provinces would include 1,751/1,870 space-time records. Moreover, the availability of more granular VCR data depends on the digitalization of immunization registries, which has been implemented asymmetrically across Italian regions. Second, while the FE model controls for time-invariant unobserved variables (e.g., different local policies), we cannot exclude the presence of time-varying omitted variables. For instance, Fallani et al. (12) concluded that in addition to the overall benefit of increasing VCR_65+_, Italian regions with a higher market share of the MF59-adjuvanted trivalent SIV showed a further decrease in P&I_65+_. However, data on the procurement of single SIV types are typically available for NUTS-2 level only and these data cover only a limited time-span. Finally, P&I_65+_ is not a laboratory-confirmed endpoint, while laboratory confirmation is the most specific outcome and is currently considered to be the gold standard in SIV efficacy and effectiveness studies (4). On the other hand, among other registry-based influenza-related outcomes [e.g., AC mortality, which is not recommended for SIV effectiveness research (4)], endpoints related to influenza and/or pneumonia are among the most specific and, at the same time, more sensitive than laboratory-confirmed influenza (31). This means that the P&I_65+_ outcome would additionally cover cases that are not tested for influenza and cases of pneumonia initially triggered by influenza virus, which is the most common complication (32). On the assumption that 10% of pneumonia deaths are initially caused by influenza, and that the true effectiveness of SIV is 50%, the expected effectiveness against mortality due to pneumonia would be only 5% (4). We must therefore acknowledge that the observed effect estimate is probably underestimated.

In conclusion, following the construction of a granular longitudinal dataset, this analysis revealed that increasing SIV VCRs yielded significant population-level benefits. National and local policy-makers and other stakeholders should implement effective strategies designed to enhance the community's demand for and access to SIV, with the final aim of achieving a minimum VCR of 75%.

## Data availability statement

The original contributions presented in the study are included in the article/Supplementary material, further inquiries can be directed to the corresponding author.

## Author contributions

AD, AO, and GI conceptualized the study. AD, DP, MO, AB, FB, DC, MC, CC, DF, EP, CR, EV, and The FluCov Study Group collected data. AD performed the data analysis and wrote the first draft of the manuscript. All authors provided edits to the manuscript draft.

## The FluCov study group

Daniela Amicizia and Federico Grammatico: Regional Health Agency of Liguria, Genoa, Italy and Department of Health Sciences, University of Genoa, Genoa, Italy; Catia Rosanna Borriello: Directorate General for Health, Lombardy Region, Milan, Italy; Francesco Chini: Directorate for Health and Social Policy, Lazio Region, Rome, Italy; Lorenza Ferrara: Regional Epidemiology Reference Service for the Surveillance, Prevention and Control of Infectious Diseases, Local Health Unit of Alessandria, Alessandria, Italy; Fabio Filippetti: Regional Health Agency, Ancona, Italy; Daniela Loconsole and Francesca Centrone: Department of Interdisciplinary Medicine, University of Bari, Aldo Moro Policlinico, Bari, Italy; Mario Palermo: Regional Epidemiological Observatory, Health Department, Sicily Region, Palermo, Italy; Simone Pizzini: Department of Public Health and Pediatrics, University of Turin, Turin, Italy.
