# Supplementary material for: Population-level benefits of increasing influenza vaccination uptake among Italian older adults: results from a granular panel model
Source: Front Public Health. 2023 Aug 3;11:1224175. doi: 10.3389/fpubh.2023.1224175 (PMC10435743; doi:10.3389/fpubh.2023.1224175)
Supplement: Supplementary file 1 [file Data_Sheet_1.docx]

Supplementary Material

Population-level benefits of increasing influenza vaccination uptake among Italian older adults: Results from a granular panel model

Alexander Domnich^1^, Andrea Orsi^1,2,3^, Donatella Panatto^2,3^, Matilde Ogliastro^2^, Alessandra Barca^4^, Fabrizio Bert^5,6^, Danilo Cereda^7^, Maria Chironna^8^, Claudio Costantino^9^, Daniel Fiacchini^10^, Elena Pariani^3,11^, Caterina Rizzo^12^, Enrico Volpe^4^, Giancarlo Icardi^1,2,3^ and The FluCoV Study Group

^1^Hygiene Unit, San Martino Policlinico Hospital - IRCCS for Oncology and Neurosciences, Genoa, Italy

^2^Department of Health Sciences (DISSAL), University of Genoa, Genoa, Italy

^3^Interuniversity Research Center on Influenza and Other Transmissible Infections (CIRI-IT), Genoa, Italy

^4^Directorate for Health and Social Policy, Lazio Region, Rome, Italy

^5^Department of Public Health and Pediatrics, University of Turin, Turin, Italy

^6^Hygiene and Infection Control Unit, ASL TO3, Turin, Italy

^7^Directorate General for Health, Lombardy Region, Milan, Italy

^8^Department of Interdisciplinary Medicine, University of Bari, Aldo Moro Policlinico, Bari, Italy

^9^Department of Health Promotion Sciences, Maternal and Infant Care, Internal Medicine and Medical Specialties (PROMISE) "G. D'Alessandro", University of Palermo, Palermo, Italy

^10^Public Health Department, AST Ancona, Italy

^11^Department of Biomedical Sciences for Health, University of Milan, Milan, Italy

^12^Department of Translational Research on New Technologies in Medicine and Surgery, University of Pisa, Pisa, Italy

*** Correspondence:**Alexander Domnich: alexander.domnich@hsanmartino.it

**Supplementary Table 1.** Availability of influenza vaccination coverage data, by year and NUTS (Nomenclature of Territorial Units for Statistics) level.

| **Region** | **NUTS level (*N* units)** | **Years (*N* units)** | ***N* space-time observations** |
| --- | --- | --- | --- |
| Piedmont | 3 (8) | 2012–2019 (8) | 64 |
| Aosta Valley | 3 (1) | 2003–2019 (17) | 17 |
| Lombardy | 3 (11)^a^ | 2003–2008, 2012–2016, 2018–2019^b^ (13) | 131 |
| Trentino-Alto Adige | 3 (2) | 2003–2019 (17) | 34 |
| Veneto | 3 (7) | 2008–2019 (12) | 84 |
| Friuli-Venezia Giulia | 3 (4) | 2003–2019^c^ (17) | 58 |
| Liguria | 3 (4) | 2010–2011, 2013–2019 (9) | 36 |
| Emilia Romagna | 3 (9) | 2003–2019^d^ (17) | 141 |
| Tuscany | 3 (10) | 2003–2019 (17) | 170 |
| Umbria | 2 (1) | 2003–2019 (17) | 17 |
| Marche | 3 (5) | 2013–2019 (7) | 35 |
| Lazio | 3 (5) | 2003–2019 (17) | 85 |
| Abruzzo | 3 (4) | 2012–2019 (8) | 32 |
| Molise | 2 (1) | 2003–2019 (17) | 17 |
| Campania | 2 (1) | 2003–2019 (17) | 17 |
| Apulia | 3 (5)^e^ | 2007–2019 (13) | 65 |
| Basilicata | 2 (1) | 2003–2019 (17) | 17 |
| Calabria | 2 (1) | 2003–2019 (17) | 17 |
| Sicily | 3 (9) | 2010–2019 (10) | 90 |
| Sardinia | 2 (1) | 2003–2019 (17) | 17 |

^a^Milan and the province of Monza-Brianza were considered as a single unit.

^b^For 2018 and 2019, data were not available for the provinces of Varese, Como, Cremona, Mantua, Lecco and Lodi.

^c^For the period 2016–2019, data were partially unavailable (2016 and 2017: missing data for the provinces of Udine and Gorizia; 2018 and 2019: missing data for the provinces of Pordenone, Trieste and Gorizia).

^d^For the period 2016–2019, data were not available for the provinces of Forlì-Cesena, Ravenna and Rimini.

^e^The provinces of Bari and Barletta-Andria-Trani were considered as a single unit.

**Supplementary Table 2** Multivariable fixed- and random-effects models on the association between pneumonia- and influenza-related mortality and influenza vaccination coverage rates among Italian older adults: Sensitivity analysis on excluding NUTS-2 observations (*N* = 379)

| **Variable** | **FE model** | | **RE model** | |
| --- | --- | --- | --- | --- |
|  | **Estimate (HAC SE)** | ***P*-value** | **Estimate (HAC SE)** | ***P*-value** |
| VCR_65+_ | -0.005 (0.001) | < 0.001^***^ | -0.006 (0.001) | < 0.001^***^ |
| P_75+_ | 0.005 (0.012) | 0.66 | -0.004 (0.011) | 0.72 |
| P_≤14_ | 0.082 (0.035) | 0.019^*^ | 0.089 (0.026) | < 0.001^***^ |
| GDP^a^ | 0.983 (0.215) | < 0.001^***^ | 1.085 (0.115) | < 0.001^***^ |
| Mort^a^ | 2.651 (0.239) | < 0.001^***^ | 2.131 (0.206) | < 0.001^***^ |
| Temp | -0.013 (0.004) | 0.001^**^ | -0.020 (0.003) | < 0.001^***^ |
| Dens^a^ | -0.321 (0.329) | 0.33 | 0.045 (0.038) | 0.24 |
| Bed^a^ | -0.032 (0.138) | 0.82 | -0.030 (0.110) | 0.78 |
| HCP^a^ | -0.089 (0.173) | 0.61 | -0.022 (0.115) | 0.85 |
| H1N1s dummy | Ref | Ref | Ref | Ref |
| H1N1pdm09 dummy | -0.008 (0.037) | 0.84 | -0.003 (0.038) | 0.93 |
| H3N2 dummy | 0.088 (0.034) | 0.010^*^ | 0.116 (0.033) | < 0.001^***^ |
| B dummy | 0.107 (0.040) | 0.007^**^ | 0.142 (0.038) | < 0.001^***^ |
| H1N1s/B dummy | -0.036 (0.049) | 0.46 | -0.015 (0.047) | 0.75 |
| H1N1pdm09/H3N2 dummy | 0.113 (0.039) | 0.004^**^ | 0.163 (0.038) | < 0.001^***^ |
| *R*^2^ | 0.484 | < 0.001^***^ | 0.522 | < 0.001^***^ |

Bed, ordinary hospital beds per 10,000 inhabitants; Dens, population density, inhabitants per km^2^; FE, fixed-effects model; GDP, gross domestic product (1,000 €) per capita based on purchasing power parity; HAC SE, heteroskedasticity and autocorrelation consistent standard errors; HCP, healthcare professionals operating in public and accredited private health facilities per 10,000 inhabitants; Mort, baseline mortality rate per 10,000 inhabitants; P_≤14_, proportion (%) of children aged ≤ 14 years in the total population; P_75+_, proportion (%) of older adults aged ≥ 75 years in the population aged ≥ 65 years; RE, random-effects model; SD, standard deviation; VCR_65+_, influenza vaccination coverage rate in subjects aged ≥ 65 years.

^a^Regression coefficients are based on log*_e_*-transformed variables.

^***^*P* < 0.001

^**^*P* < 0.01

^*^*P* < 0.05.
